# Degradation of Orange G, Acid Blue 161, and Brillant Green Dyes Using UV Light‐Activated GA–TiO_2_–Cd Composite

**DOI:** 10.1002/gch2.202300271

**Published:** 2024-04-14

**Authors:** Derya Tekin, Derya Birhan, Taner Tekin, Hakan Kiziltas

**Affiliations:** ^1^ Department of Metallurgy and Materials Engineering Ataturk University Erzurum 25240 Turkey; ^2^ Department of Chemical Engineering Ataturk University Erzurum 25240 Turkey

**Keywords:** graphene aerogel, hydrothermal, metal doping, photocatalytic activity, TiO_2_ nanoparticles

## Abstract

In this study, photocatalysts with high photocatalytic activity performance are synthesized by synthesizing graphene aerogel‐supported, cadmium‐doped TiO_2_ composites by hydrothermal method for the effective degradation of organic dyes in wastewater. Here, GA–TiO_2_–Cd is investigated as a photocatalyst for the degradation of toxic dyes named Orange G, Acid Blue 161, and Brilliant Green in the UV part of the light spectrum. As a result of the experiments, it is observed that the effective decomposition of organic dyes is due to graphene aerogel (GA) and cadmium‐doped TiO_2_ nanoparticles. The results show that for 20 ppm solutions of Orange G, Acid Blue 161, and Brilliant Green, dyes are removed at approximately 81.075%, 84.15%, and 95.18% in 120 min. The morphology and elemental analysis of the synthesized composites are determined using SEM‐EDS, crystal structure analysis by XRD, chemical bond analysis by FTIR, optical properties by UV‐Vis‐NIR spectrophotometry, and thermal resistance by TGA analysis.

## Graphical abstract

TiO_2_, a semiconductor, is widely used today due to its economy, nontoxicity, and photocatalytic activity. However, agglomeration of TiO_2_ and rapid recombination of electron–hole pairs limits the photocatalytic activity. TiO_2_ photocatalysts are developed that exhibit superior photocatalytic activity under UV light. Doping with transition metals causes the properties of TiO_2_ to change.

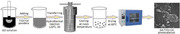

## 1 Introduction

Wastes from the paper, plastic, and textile industries are discharged into water sources without any pretreatment. These wastes often contain organic dyes that are not biodegradable and cause serious problems for the environment.^[^ ^1^ ^]^ Since organic dyes used in the industry are resistant to biodegradation and have the ability to remain insoluble in water, even a small amount of dye discharge affects human and marine life negatively.^[^ ^2^ ^]^ Therefore, many low‐cost methods are needed to remove impurities from water and the environment.^[^ ^3^ ^]^ To date, many methods such as biological decomposition, chemical oxidation, and physical adsorption have been used to remove organic dyes from water. It has been determined that ozone degradation,^[^ ^4^ ^]^ electrochemical degradation,^[^ ^5^ ^]^ activated carbon,^[^ ^6^ ^]^ membrane filtration,^[^ ^7^ ^]^ adsorption,^[^ ^8^ ^]^ and photocatalysis are effective methods of removing pollution.^[^ ^9^ ^]^ Photocatalysis is a purification process to degrade organic dyes and reduce the concentration of metal ions by stimulating the oxide layer on the surface under sunlight.^[^ ^10^ ^]^ Under the influence of sunlight, electrons in the conduction band and holes in the valence band are formed, revealing the photocatalytic ability of catalysts. These cavities are carried to the catalyst surfaces and form radicals (OH^•^, $O_{2}^{•-}$) that oxidize toxic or active pollutants.^[^ ^11^ ^]^ In recent years, semiconductor photocatalytic technology has been used to separate organic pollutants from wastewater. TiO_2_, one of the metal oxide semiconductors, is widely used due to its low cost, non‐toxicity, high chemical stability, photo corrosion resistance, and photocatalytic activity.^[^ ^12^, ^13^ ^]^ However, the agglomeration of TiO_2_ and the rapid recombination of electron–hole pairs limit photocatalytic activity.^[^ ^14^, ^15^ ^]^ Various methods have been developed for the development of TiO_2_‐based photocatalysts that exhibit high activity under both UV and visible light. Some of the bandgap narrowing methods do not show high photoactivity and stability for long‐term use.^[^ ^16^ ^]^ The addition of transition metal ions with incomplete subshell structures to the TiO_2_ lattice causes changes in the crystal size, crystal structure, and electronic properties of TiO_2_. The crystal structure of TiO_2_ may change due to the difference in charge and radius between Ti^+4^ and metal ions. Metal ions inhibit the growth of the crystal, causing the radius to shrink. As a result, the light absorption of TiO_2_ increases and the bandgap energy decreases.^[^ ^17^, ^18^, ^19^ ^]^ Doping of TiO_2_ nanoparticles with transition metals to form heterostructures and nanocomposites improves photocatalytic performance. In the doping method, it is proposed to increase the charge collection efficiency by creating areas that will increase the absorption of light.^[^ ^20^ ^]^

Therefore, doping with transition metals such as Cu, Ni, Zn, Co, Fe, and Cd caused the properties of TiO_2_ to change for certain and desired applications.^[^ ^21^, ^22^ ^]^ TiO_2_/Cd structures produced by the electrospinning technique were used as a photocatalyst in the removal of methylene orange dye after the calcination process. As a result of the experiments, it was reported that Cd‐doped TiO_2_ nanoparticles showed faster photocatalytic efficiency than pure TiO_2_ nanoparticles.^[^ ^23^ ^]^ Agglomeration of nanosized TiO_2_ particles, which exhibit superior photocatalytic performance, causes loss of active site and decreased photocatalytic activity. In recent years, 3D graphene aerogel (GA) has been studied to overcome agglomeration.^[^ ^24^, ^25^ ^]^ 3D graphene aerogel with meso and micropores attracts great attention in the field of photolysis due to its superior mechanical properties, high electrical conductivity, excellent optical transmittance, large specific surface area, and lightness.^[^ ^26^ ^]^ Graphene aerogel with hydrophobic properties is an ideal support material for photocatalysts used in wastewater treatment and facilitates the separation of graphene‐supported photocatalysts from aqueous phases. In addition to its high hydrophobicity, two properties are important for the photodegradation of wastewater; i) it is highly transparent as it transmits only 2–3% of visible light; ii) it can increase photocatalytic activity as it prevents recombination of electron–hole pairs thanks to its conductive property. For example, Hou et al. synthesized P25‐graphene hydrogels and removed methylene blue under UV irradiation.^[^ ^27^ ^]^ Cui et al. synthesized TiO_2_‐rGH hydrogels for Cr(IV) removal under UV light.^[^ ^28^ ^]^ Ahmad and Majid in a study conducted by GO, it was determined that due to its large surface area, GO creates a more active surface area and increases the photocatalytic degradation of organic dyes. CdO structures with 3.3% GO removed 95% of the MB dye in only 35 min.^[^ ^29^ ^]^ Oh et al. prepared a porous SiO_2_/CdO–graphene composite using the self‐assembly method. SiO_2_/CdO–graphene composite has a small bandgap and a porous structure, and it was found that the photocatalytic activity of organic dyes increased under visible light.^[^ ^30^ ^]^

In this study, GA support material and TiO_2_ nanoparticles were combined with Cd metal ions and synthesized by hydrothermal method. Morphology and crystal structure analyses of the synthesized photocatalysts were examined using SEM and XRD, optical properties were examined using UV–Vis–NIR spectrophotometry, and chemical composition and bond analyses were examined using FTIR. The thermal resistances of the photocatalysts were determined by TGA analysis. The photocatalytic activity of the photocatalysts will be tested using UV‐spectroscopy at wavelengths of 578, 624, and 475 nm using Acid Blue 161, Brilliant Green, and Orange G dyes.

## 2 Experimental Section

### 2.1 Materials

Acetic acid (CH_3_CO_2_H, 99.7%) Merck, cadmium nitrate (Cd(NO_3_)_2_.4H_2_O>99.9%), titanium (IV) tetraisopropoxide (TTIP) (Ti[OCH(CH_3_)_2_]_4_, 97%), ethanol (C_2_H_5_OH, 99.8%), 2‐propanol ((CH_3_)_2_CHOH, 99.5%), sulfuric acid (H_2_SO_4_, 95–97% by weight), nitric acid (HNO_3_, 65%), graphite powders (<45 µm, 99.99%), potassium permanganate (KMnO_4_, 99%), sodium nitrate (nano_3_, 99%), and hydrogen peroxide (H_2_O_2_, 30%) were purchased from Sigma Aldrich. For the photocatalytic experiment, Acid Blue 161 (C_20_H_13_N_2_O_5_SNaCrx 100%) Sigma‐Aldrich, Orange G (C_16_H_10_N_2_Na_2_O_7_S_2_ 100%) Fluka and Brillant Green (C_27_H_34_N_2_O_4_S 100%) were purchased from Acros Organics.

### 2.2 Synthesis of Graphene Oxide

Graphene oxide was synthesized according to the Hummer method. First, 69 mL of sulfuric acid, 3 g of graphite powder, and 1.5 g of NaNO_3_ were mixed in a 250 mL flask. It was placed in an ice bath to reduce the solution temperature to 0 °C. Then 9 g of KMnO_4_ was slowly added to the solution. Thereafter, the solution come to room temperature, 138 mL of the ionized water was added and stirred at 98 °C for 15 min. After stirring for 15 min, 480 mL of deionized water and H_2_O_2_ (30%) solution were added. The resulting graphene oxide was washed several times with water and ethanol and dried at 60 °C.

### 2.3 Synthesis of Graphene Aerogel

1.25 g of GO particles in 100 mL of ionized water were sonicated for 1 h. Subsequently, the mixture was transferred into a 300 mL Teflon‐lined stainless steel autoclave and heated at 180 °C for 15 h. The resulting graphene aerogel was freeze‐dried for 1 d. Finally, it was dried at 105 °C.

### 2.4 Synthesis of TiO_2_ Nanoparticles

10 mL of titanium tetraisopropoxide was dissolved in 70 mL of 2‐propanol for 15 min. The solution was refluxed at 80 °C for 1 h. Nitric acid was added to adjust the pH of the solution (pH = 1.5). After reflux, the solution was filtered, dried, and calcined at 500 °C for 3 h in a muffle furnace.

### 2.5 Synthesis of TiO_2_–Cd Photocatalyst

50 mL of ethanol and 30 mL of titanium tetraisopropoxide were stirred for 30 min. On the other hand, 1.817 g of Cd(NO_3_)_2_.4H_2_O was dissolved in 50 mL of ethanol and 25 mL of 0.1 m acetic acid. The Cd solution was added to the Ti solution and heated in an autoclave at 150 °C for 8 h. In the final step, the white precipitate was filtered, dried, and calcined at 500 °C for 3 h.

### 2.6 Synthesis of GA–TiO_2_–Cd Photocatalyst

GA–TiO_2_–Cd photocatalyst was synthesized by hydrothermal method. 0.2 g of GO was dissolved in 80 mL of ethanol and 80 mL of DI by ultrasonic treatment, and then 1.1 g of Cd–TiO_2_ powders were added and mixed for another 1 h to obtain a homogeneous solution. Finally, the solution was transferred to a 300 mL stainless steel Teflon autoclave and heated at 120 °C for 3 h. The prepared solution was filtered and dried.

### 2.7 Characterization

The chemical bond structures of the synthesized composites were examined using the transmittance mode with Fourier transform infrared spectroscopy (FTIR, VERTEX 70V) in the range of 4000–500 cm^−1^. Crystal structure analysis was determined by X‐ray diffractometry (XRD, PANalytical Empyrean) using Cu‐Kα (1.5418 Å) radiation between 10 and 90°, and morphology analysis were determined by scanning electron microscope (SEM, Zeiss Sigma 300). Energy dispersive X‐ray spectroscopy (EDS, Zeiss Sigma 300) was used for the quantitative analysis of the elements. The optical properties were determined using a UV–Vis–NIR spectrophotometer (Shimadzu UV‐3600 Plus) in transmittance mode between 200 and 800 nm wavelength. The thermal analysis of the composites was investigated in an air atmosphere with a heating rate of 10 K min^−1^ using an aluminum pan at 28–1000 °C using the 409PC/PG model (NETZSCH STA).

### 2.8 Degradation Experiments

For the photocatalytic degradation study, three dyes were selected such as Orange G, Acid Blue 161, and Brillant Green. To eliminate the maximum dye level, a Pen‐Ray UV (ColeParmer) lamp with a wavelength of 254 nm and a light intensity of 44 W m^−2^ was used. The saturated oxygen concentration in the solution medium was achieved with the help of a pump. Experiments were made by adding 100 mg of photocatalyst to a 20 mg L^−1^ dye solution with a volume of 400 mL. Before starting the experiments, they were mixed for 30 min in the dark for the adsorption and desorption of dye molecules on the surface of the photocatalysis. Samples taken at different time intervals were centrifuged to determine the concentration. The concentration of the samples was measured at wavelengths of 578, 624, and 475 nm using a UV spectrophotometer (Optizen α). **Table** **1** shows the molecular properties of Brilliant Green, Acid Blue 161, and Orange G dyes. Decomposition percentages of Acid Blue 161, Brilliant Green, and Orange G dyestuffs before and after photocatalytic degradation were calculated using Equation 1.

(1) $$\mathrm{Percentage}\;\mathrm{of}\;\mathrm{degradation}\;\%=\left(C_{0}-C_{s}/C_{0}\right)\times 100\;\;$$

*C*_0_ and *C*_s_ represent the initial and final dye concentrations in the aqueous phase, respectively.

**Table 1:** Molecular properties of Brillant Green, Acid Blue 161 and Orange G dyes.

Dye name	*ƛ*_max_	Molecular weight [g mol^−1^]	Structure	Chemical formula
Brillant Green	624	482.6	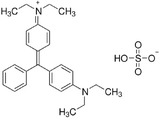	C_27_H_34_N_2_O_4_S
Acid Blue 161	578	394.40	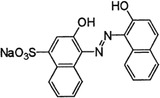	C_20_H_13_N_2_O_5_SNaCrx
Orange G	475	452.38	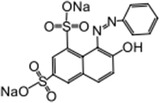	C_16_H_10_N_2_Na_2_O_7_S_2_

## 3 Results and Discussion

### 3.1 Scanning Electron Microscopy (SEM)

**Figure 1:**
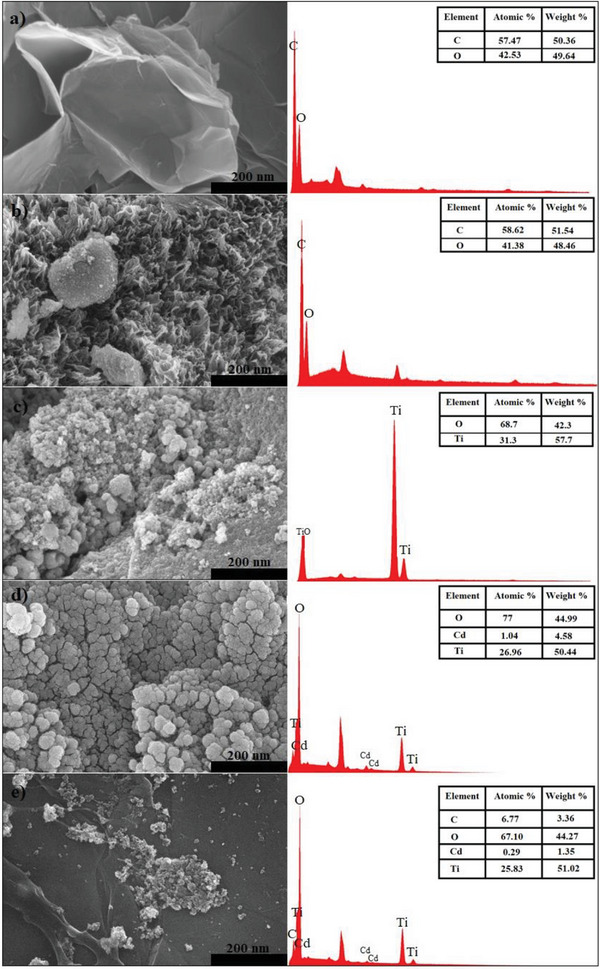
SEM‐EDS images of GO, GA, TiO_2_, TiO_2_–Cd and GA–TiO_2_–Cd photocatalysts.

SEM‐EDS images of the synthesized photocatalysts are shown in **Figure** **1**. In the SEM image in Figure 1a, it is seen that GO has a thin and wrinkled structure that comes together randomly. GO layers become unstable due to the oxidation process. Graphite sheets have been successfully synthesized into a large number of GO sheets.^[^ ^31^ ^]^ The SEM image shown in Figure 1b reveals that the GA retains its wrinkled and 3D structure. It is clearly seen that the graphene aerogel structure has a honeycomb‐like structure with different pore sizes.^[^ ^32^ ^]^ In Figure 1c, it is seen that the TiO_2_ nanoparticles are spherical in size and uniformly dispersed. The large particles shown in the figure are thought to be formed by the clustering of small particles. In the SEM image of the TiO_2_–Cd composite shown in Figure 1d, it is seen that the agglomeration of TiO_2_ nanoparticles started to break down after the addition of Cd metal ions. Many researchers have reported that adding metal–nonmetal additives to semiconductor materials will reduce agglomeration.^[^ ^33^ ^]^ Therefore, obtaining the specific surface area and low particle size in the study is the precursor of high photocatalytic activity. In the SEM image of the GA–TiO_2_–Cd composite shown in Figure 1e, it is seen that sufficient interfacial contact is provided between the TiO_2_–Cd composite and the GA layers. This contact between the GA layer and the metal particles is suitable for effective charge transfer. SEM images of the TiO_2_–Cd composite and GA–TiO_2_–Cd composite have different surface morphology, indicating that the GA–TiO_2_–Cd composite was synthesized successfully. EDS spectra of GO, GA, TiO_2_, TiO_2_–Cd, and GA–TiO_2_–Cd composites confirmed the presence of C, O, Ti, and Cd elements, as shown in Figure 1. It should be noted that the GA–TiO_2_–Cd composite is higher in terms of Ti content compared to the TiO_2_–Cd composite.

### 3.2 X‐Ray Diffraction (XRD)

**Figure 2:**
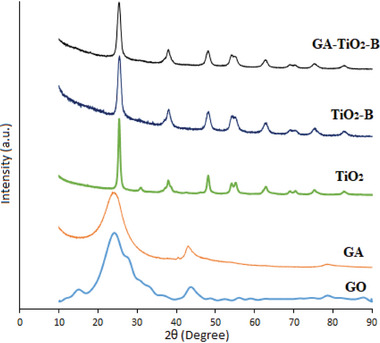
XRD plot of GO, GA, TiO_2_, TiO_2_–Cd and GA–TiO_2_–Cd photocatalysts.

**Figure** **2** shows the XRD models of GO, GA, TiO_2_, TiO_2_–Cd, and GA–TiO_2_–Cd composites. Due to the presence of oxygen groups on the graphene nanolayers, GO shows a diffraction peak at 11° corresponding to the (002) plane. This peak indicates the presence of oxygen and water between the graphite layers after the oxidation of GO. TiO_2_ nanoparticles show a diffraction peak corresponding to the anatase phase at the 25.3 (101) plane and the rutile phase at the 27.4 (110) plane. The diffraction peaks confirmed the presence of the anatase phase of the TiO_2_ nanoparticles (JCPDS No: 21‐1272).^[^ ^34^ ^]^ TiO_2_ nanoparticles calcined at 500 °C show the diffraction peaks of the anatase phase with tetragonal structure at 2*θ* = 25.90, 47.81, 52.85, 56.07, and 61.99°.^[^ ^35^ ^]^ The TiO_2_–Cd composite synthesized by the hydrothermal method shows the crystal structure of the anatase phase at 2*θ* = 25.49, 37.79, and 48.04°. XRD analysis showed that the anatase phase was successfully synthesized and no peaks of the rutile phase were formed. Studies in the literature reported that the anatase phase showed higher photocatalytic activity compared to other phases.^[^ ^36^ ^]^ Since CdO is in the amorphous phase and there is a very low Cd concentration in the composite, no peak of CdO was detected. Although the addition of Cd metal ions shows the characteristics of TiO_2_ anatase, the decrease in the intensity of the anatase peaks proves the presence of Cd atoms. In the graphene aerogel‐supported TiO_2_–Cd composite, the intensity of the diffraction peaks was slightly decreased due to the graphene aerogel additive. A small peak is observed in the GA–TiO_2_–Cd composite, corresponding to the (002) plane of the graphene aerogel at 25.1°. The peaks seen in the (101), (105), and (204) planes confirm the anatase phase of TiO_2_ (JCPDS No: 21‐1272). The graphene aerogel showed a weak peak due to the small particle size, low amount, and homogeneous distribution. All these results confirm that the GA–TiO_2_–Cd composite was successfully synthesized.

### 3.3 Fourier Transform Infrared Spectroscopy (FTIR)

**Figure 3:**
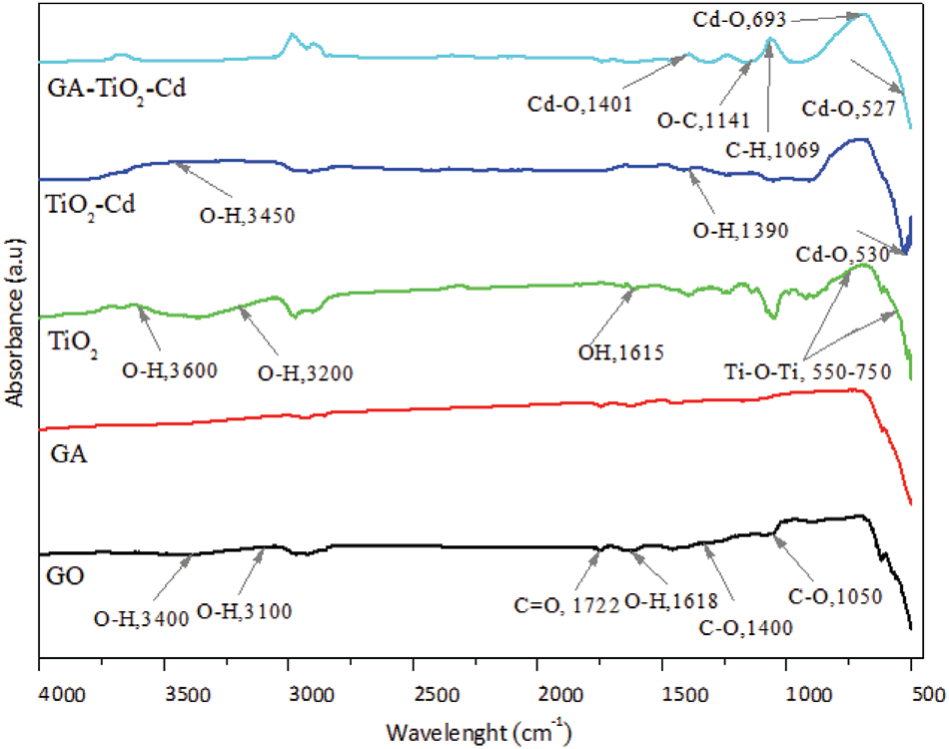
FTIR spectrum of GO, GA, TiO_2_, TiO_2_–Cd and GA–TiO_2_–Cd photocatalysts.

**Figure** **3** shows the FTIR spectra of GO, GA, TiO_2_, TiO_2_–Cd, and GA–TiO_2_–Cd composites. Analysis was performed in the range of 4000–500 cm^−1^ to determine the surface functional groups. The peak of the GO nanolayers corresponding to the wavelengths at 3100 and 3400 cm^−1^ shows the adsorbed water molecules and the O─H groups in the GO structure. In addition, the peaks at 1618 and 1722 cm^−1^ correspond to the O─H bending and C═O stretching of the carbonyl group, respectively, and the bands at 1050 and 1400 cm^−1^ correspond to the C─O stretching vibration.^[^ ^37^ ^]^ When the peaks of GA are compared with GO, it is seen that the oxygen functional groups in the structure of GO disappear during the synthesis of GA and the GO layers decrease.^[^ ^38^, ^39^ ^]^ The absorption band and Ti─O─Ti bending vibrations were observed between 550 and 750 cm^−1^ wavelengths, confirming the crystal structure of TiO_2_. The sharp peaks seen at 1615 and 1625 cm^−1^ are attributed to the characteristic bending vibrations of the OH group.^[^ ^40^ ^]^ The absorption peak observed between 3200 and 3600 cm^−1^ is due to the interaction of the hydroxyl OH group in the water structure. In the analysis of the TiO_2_–Cd composite, Cd‐doped TiO_2_ nanoparticles exhibit a vibrational band of O─H functional groups at 1390 and 3450 cm^−1^ wavelengths. O─H functional groups enhance the photocatalytic activity by generating hydroxyl radicals by absorbing ─OH in wastewater and via electrons in the conduction band of the semiconductor catalyst. The vibration band at 530 cm^−1^ is due to the presence of Cd─O and Ti─O bands of metal oxides in the composite structure.^[^ ^41^ ^]^ The vibration band seen at 2350 cm^−1^ due to the calcining process is assigned to the physical adsorption of CO and CO_2_. In the GA–TiO_2_–Cd composite, strong and sharp peaks at 527, 693, and 1401 cm^−1^ correspond to the Cd─O stretching vibration. The peaks at 1069, 1141, and 1645 cm^−1^ show the stretching vibrations of the functional groups containing C─H, O─C, and oxygen in the graphene aerogel structure. Analysis results confirm the synthesis of GA–TiO_2_–Cd composite.

### 3.4 Thermal Gravimetric Analysis (TGA)

**Figure 4:**
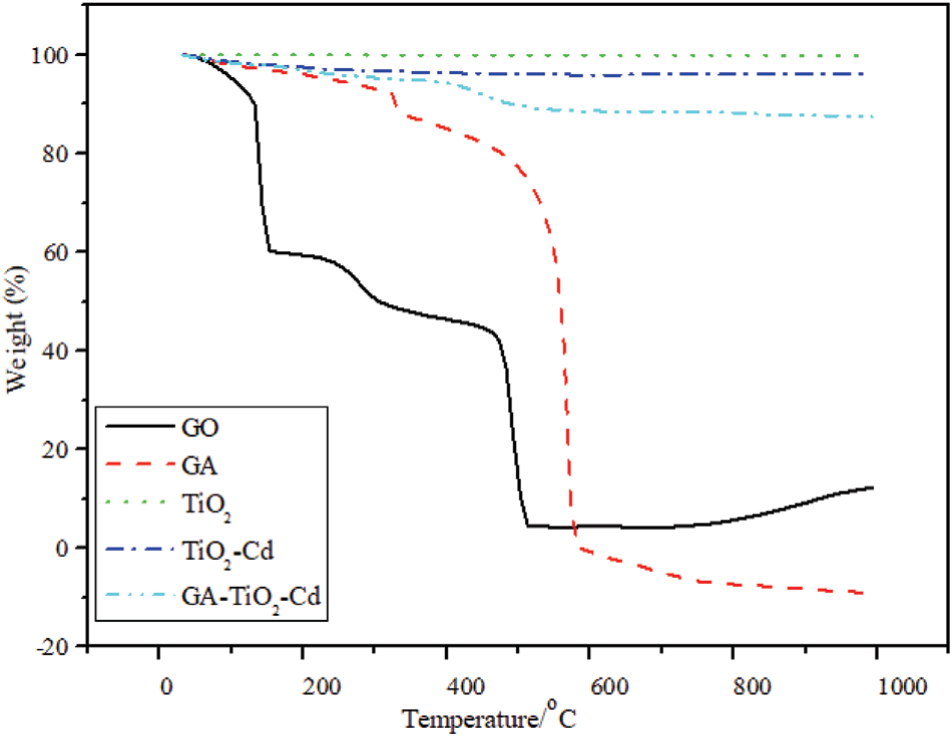
TGA plot of GO, GA, TiO_2_, TiO_2_–Cd and GA–TiO_2_–Cd photocatalysts.

**Figure 5:**
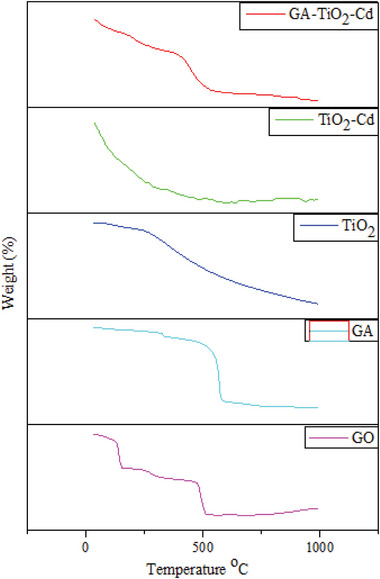
TGA curves of photocatalysts.

**Figures** **4** and **5** shows the TGA curves of GO, GA, TiO_2_, TiO_2_–Cd, and GA–TiO_2_–Cd composites that weight loss in the air atmosphere. The first mass loss of GO occurred between 120 °C and 300 °C with the removal of the absorbed water. The second mass loss occurs at 500 °C. The percent weight loss of GO during this period is 87.71%.^[^ ^42^ ^]^ Two‐stage mass loss is seen in the TGA analysis of GA. The first mass loss occurs between 100 °C and 250 °C with the removal of oxygen groups in the structure during the reduction process of GO. The second mass loss occurs between 250 °C and 400 °C by the decomposition of GO molecules.^[^ ^43^ ^]^ The percent weight loss of GA is 109.04%. In the TGA analysis of TiO_2_ in powder form, the weight loss between 80 °C and 115 °C is due to the evaporation of moisture in the structure. TiO_2_ nanoparticles are stable up to 750 °C. The weight loss percent of TiO_2_ is 0.41%. TiO_2_ nanoparticles show thermal stability due to their high thermal resistance and carboxyl groups in the structure.^[^ ^44^ ^]^ In the TGA analysis of the TiO_2_–Cd composite, weight loss occurred at a temperature of around 345 °C due to the decomposition of the ─OH groups in the structure. After this temperature, no peak was observed as the compound remained intact.^[^ ^45^ ^]^ The weight loss percent of TiO_2_–Cd composite is 3.90%. Three different mass losses occur in the TGA curve of the GA–TiO_2_–Cd composite. The first weight loss corresponds to the desorption of water in the structure at 100 °C. The second weight loss is due to the separation of oxygen‐containing functional groups from the structure between 200 and 250 °C. According to TGA analysis results, the thermal stability of GA–TiO_2_–Cd composite is higher than that of pure GO. The reason for this is that TiO_2_ in the structure of the GA–TiO_2_–Cd composite provides thermal stability.^[^ ^46^ ^]^ The percent weight loss of the GA–TiO_2_–Cd composite is 12.56%.

### 3.5 UV–Vis–NIR Spectrometer

**Figure 6:**
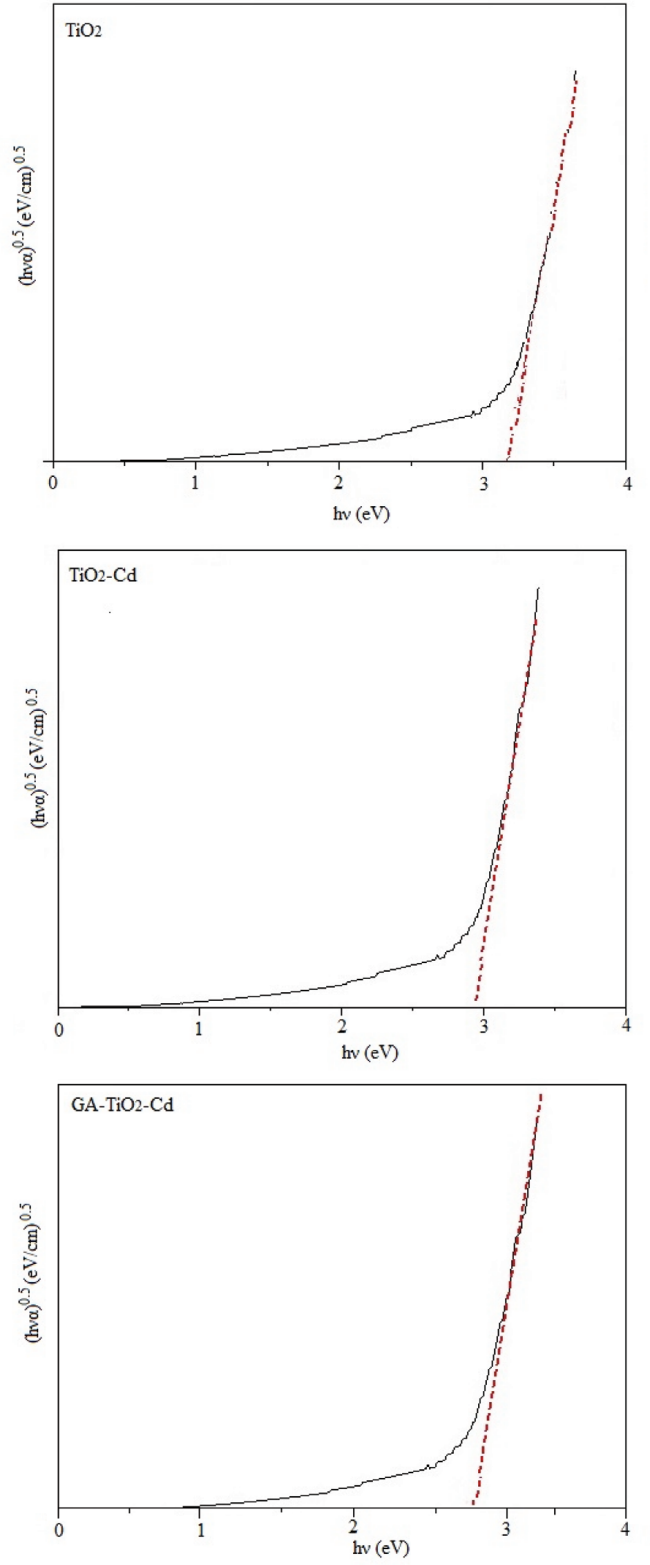
Optical bandgap of TiO_2_, TiO_2_–Cd, and GA‐TiO_2_–Cd composites.

UV–Vis–NIR spectrophotometer results of TiO_2_, TiO_2_–Cd, and GA–TiO_2_–Cd composite are shown in **Figure** **6**. To determine the estimated bandgaps of the composites, spectra were recorded using Transmittance mode at wavelengths of 200–800 nm. It was determined that the permeability (T%) of TiO_2_–Cd and GA–TiO_2_–Cd composites decreased. It can be seen that pure TiO_2_ nanoparticles show an absorption band at 356 nm. In addition, the TiO_2_–Cd composite showed absorption bands at 374 nm and the GA–TiO_2_–Cd composite showed absorption bands at 380 nm. UV–Vis–NIR spectra of the synthesized composite structures were examined separately for each sample and shown in Figure 6. To estimate the bandgap energy of TiO_2_‐based composite structures annealed at 500 °C, it was calculated using Tauc plots and Kubelka‐Munk (K‐M) formula, as shown in Figure 6. Optical bandgaps of TiO_2_, TiO_2_–Cd, and GA–TiO_2_–Cd composites were calculated together with Tauc graphs as given in Equation 2.

(2) $$\alpha h\vartheta \;=\;A{\left(h\vartheta -Eg\right)}^{n}$$

where, all the parameters have a meaning and the n exponent depends on the transition type. While *n* = 0.5 is used for the direct bandgap, *n* = 2 is used for the indirect bandgap. *E_g_* was determined as the optical energy of the composites, A as the disorder parameter constant, *h*ϑ (eV) as the energy of the photon, and α (cm^−1^) as the absorption coefficient. The optical bandgap energy of the composites was estimated by a straight line fit of *h*ϑ versus (*h*ϑα)^0.5^. Accordingly, the optical bandgap energies of TiO_2_, TiO_2_–Cd, and GA–TiO_2_–Cd composites were obtained as 3.20, 2.90, and 2.70 eV, respectively.

The mechanism diagram for the degradation carried out by composite catalysts is given below.

(3) $$\mathrm{Ti}O_{2\;}+h\vartheta \;\to \mathrm{Ti}O_{2}\;\left(e^{-}+h^{+}\right)$$

(4) $$\mathrm{Ti}O_{2}-\mathrm{Cd}+h\vartheta \to \mathrm{Ti}O_{2}-\mathrm{Cd}\;\left(e^{-}+h^{+}\right)$$

(5) $$\mathrm{Ti}O_{2}\left(e^{-}\right)+\mathrm{Cd}\left(e^{-}\right)+\mathrm{GA}\to \mathrm{GA}\left(e^{-}\right)+\mathrm{Ti}O_{2}+\mathrm{Cd}$$

(6)

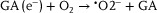



(7)

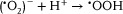



(8)

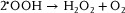



(9)

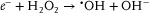

(10) $$h+OH^{-}\to {\mathrm{OH}}^{•}$$

(11) $$h^{+}+H_{2}O\to {\mathrm{OH}}^{•}$$

(12) $$\mathrm{BG}-\mathrm{AB}161-\mathrm{OG}+\mathrm{OH},h^{+}\to CO_{2}+H_{2}O$$

(13) $$\mathrm{BG}-\mathrm{AB}161-\mathrm{OG}+{O\;}_{2}^{-}\to CO_{2}+H_{2}O$$

### 3.6 Photocatalytic Degradation of Organic Dyes

**Figure 7:**
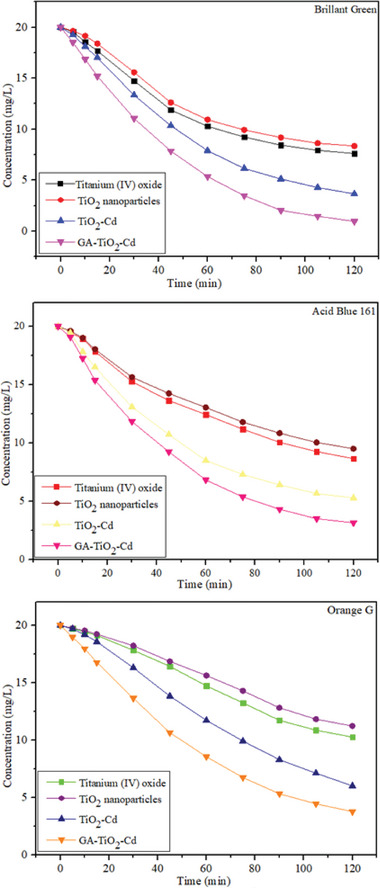
Photocatalytic degradation plot of photocatalysts on Brillant Green, Acid Blue 161 and Orange G dyes.

**Figure 8:**
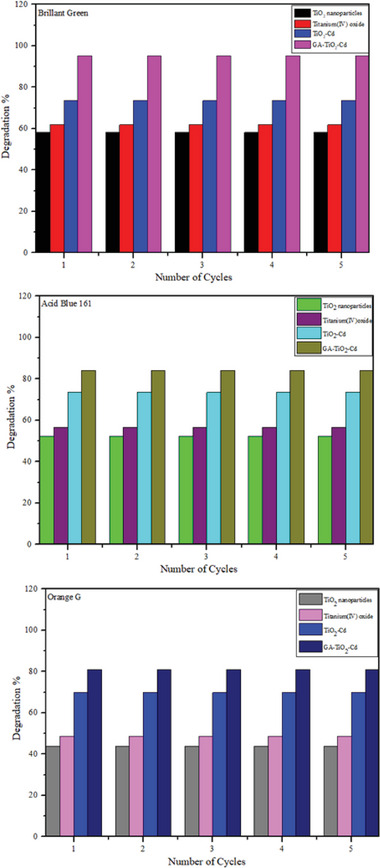
Recyclability of the synthesized photocatalysts.

**Figure 9:**
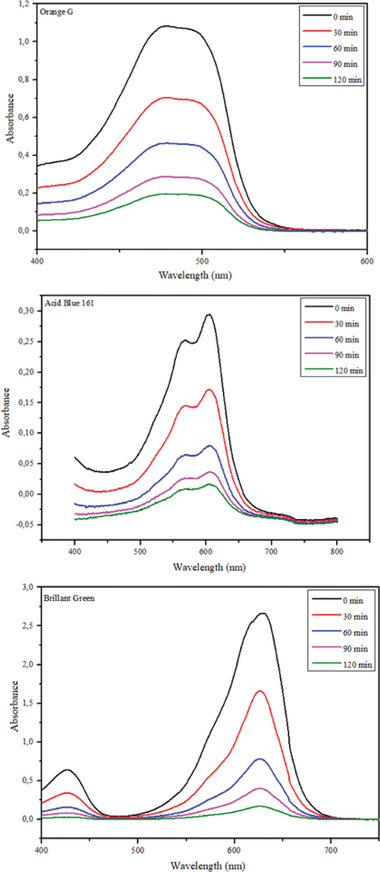
UV–Vis spectra of photocatalytic degradation of Orange G, Acid Blue 161, and Brilliant Green using GA–TiO_2_–Cd composite at different time intervals.

The photocatalytic performance of the prepared composites was determined by examining the photodegradation of organic dyes Acid Blue 161, Brilliant Green, and Orange G under UV light irradiation. **Figure** **7** shows the degradation graphs of Brilliant Green, Acid Blue 161, and Orange G dyestuffs over 120 min. Figure 7a shows the degradation of TiO_2_ nanoparticles, commercial TiO_2_, TiO_2_–Cd, and GA–TiO_2_–Cd composites on Brilliant Green dyestuff. Within 120 min, TiO_2_ nanoparticles were removed at 58.185%, commercial TiO_2_ at 61.875%, TiO_2_–Cd composite at 81.71%, and GA–TiO_2_–Cd composite at 95.18%. Degradation results show that the modification of TiO_2_ and Cd metal ions with graphene aerogel eliminates the disadvantages of TiO_2_ and provides a high photocatalytic activity. Figure 7b shows the degradation of TiO_2_ nanoparticles, Commercial TiO_2_, TiO_2_–Cd, and GA–TiO_2_–Cd composites on Acid Blue 161 dyestuff. Within 120 min, TiO_2_ nanoparticles were removed at 52.42%, commercial TiO_2_ at 56.65%, TiO_2_–Cd composite at 73.51%, and GA–TiO_2_–Cd composite at 84.15%. Photodegradation results under UV irradiation after 120 min confirmed that the GA‐supported TiO_2_–Cd composite showed more photocatalytic activity than the other samples. When TiO_2_–Cd and GA–TiO_2_–Cd composites are compared, it is seen that GA loading increases the degradation rate from 73.51% to 84.15%, because the GA doping significantly improves the photocatalytic performance by effectively increasing the charge separation on the interface. Figure 7c shows the degradation of TiO_2_ nanoparticles, commercial TiO_2_, TiO_2_–Cd, and GA–TiO_2_–Cd composites on Orange G dyestuff. Within 120 min, TiO_2_ nanoparticles were removed at 43.82%, commercial TiO_2_ at 48.64%, TiO_2_–Cd composite at 69.86%, and GA–TiO_2_–Cd composite at 81.075%. The synthesized samples showed the best photocatalytic activity on Brilliant Green dyestuff. The reason for this is that there are more ^•^OH radicals in the active sites of the photocatalysts in the Brilliant Green dyestuff compared to other dyes and higher ^•^OH radical formation occurs. The recyclability of the synthesized composites and the UV–Vis spectra of the photocatalytic degradation of GA–TiO_2_–Cd composite Orange G, Acid Blue 161, and Brilliant Green are shown in **Figures** **8** and **9**. The results obtained from the recyclability of the synthesized photocatalysts are shown in Figure 8. After each experiment, the photocatalysts were separated from the dye medium by filtering, washed, and dried. The photocatalysts were recycled five times and it was understood that their reusability was possible after recycling.

## 4 Conclusion

In this study, photocatalysts with high photocatalytic activity performance were synthesized by synthesizing graphene aerogel‐supported TiO_2_–Cd composites by hydrothermal method for the effective degradation of organic dyes in wastewater. TiO_2_ nanoparticles were doped with cadmium (Cd) to activate photocatalysts in the UV part of the light spectrum. Characterization analyses were performed using SEM‐EDS, XRD, FTIR, and TGA. According to the TGA results, the GA–TiO_2_–Cd composite suffered a weight loss of 12.56%. According to UV–Vis–NIR results, with the doping of TiO_2_ nanoparticles, the optical bandgap from 3.20 eV decreased to 2.90 eV and 2.70 eV. The photocatalytic activity of the Cd‐doped graphene aerogel–TiO_2_ composite was investigated by the degradation of Orange G, Acid Blue 161, and Brilliant Green. Under 120 min of UV light irradiation, the GA–TiO_2_–Cd composite removed 95.18% of Brilliant Green dyestuff, 84.15% of Acid Blue 161, and 81.075% of Orange G.

## Conflict of Interest

The authors declare no conflict of interest.

## Data Availability

The data that support the findings of this study are available from the corresponding author upon reasonable request.
